# Differences in pharmacokinetics of apple polyphenols after standardized oral consumption of unprocessed apple juice

**DOI:** 10.1186/s12937-015-0018-z

**Published:** 2015-04-01

**Authors:** Jürgen Wruss, Peter Lanzerstorfer, Stefan Huemer, Markus Himmelsbach, Harald Mangge, Otmar Höglinger, Daniel Weghuber, Julian Weghuber

**Affiliations:** 1 University of Applied Sciences Upper Austria, Stelzhamerstrasse 23, A-4600 Wels, Austria; 2 Johannes Kepler University, Institute for Analytical Chemistry, Linz, Austria; 3 Clinical Institute for Medical and Chemical Laboratory Diagnosis, Medical University Graz, Graz, Austria; 4 BioTechMed-Graz, University Graz, Graz, Austria; 5 Department of Pediatrics, Paracelsus Medical University, Salzburg, Austria; 6 Obesity Research Unit, Paracelsus Medical University, Salzburg, Austria

**Keywords:** Apple juice, Polyphenolics, Pharmacokinetics, Antioxidant capacity

## Abstract

**Background:**

Polyphenols are chemical compounds of the secondary plant metabolism. High concentrations can be found in various fruits including apples, berries and grapes. Polyphenols are associated with numerous health beneficial effects including a reduced risk for cardiovascular disease or diabetes. The human body cannot synthesize or store polyphenols and relies on continuous replenishment by daily diet. Unfortunately, knowledge on absorption, metabolization and excretion is still limited. The aim of this study was to determine the pharmacokinetic fate of apple polyphenols in young healthy adults.

**Methods:**

Volunteers consumed 500 mL of an unfiltered apple juice. Blood and urine samples were collected within a time period of ten hours and analyzed for their total phenolic content, concentration of selected individual polyphenolic compounds and antioxidant capacity.

**Results:**

Large differences in apple polyphenol pharmacokinetics between single subjects were observed. Those could be divided into subgroups according to fast or slow rates of polyphenol metabolism. Some subjects showed no detectable metabolism within the study time frame at all. An increase in the total phenolic content over time did not correlate with an observed, highly elevated antioxidant capacity (AOC) in the blood plasma after apple juice consumption. The determined increase of the AOC was rather a result of a high fructose content of the apple juice. No differences in renal excretion were detected between female and male subjects. However, relative concentrations were slightly higher in male subjects.

**Conclusions:**

Apple derived polyphenols can be readily detected in human blood and urine after juice consumption. The existence of sub-populations with different pharmacokinetics suggests significant variations in the individual metabolism rates of polyphenolic substances with implications on bioavailability and potential health effects within the body.

**Trial registration:**

O2413 (Ethics Commissions of Upper Austria) and 415-EP/73/233-2013 Salzburg (Ethics Commissions of Salzburg).

**Electronic supplementary material:**

The online version of this article (doi:10.1186/s12937-015-0018-z) contains supplementary material, which is available to authorized users.

## Introduction

Phytochemicals such as polyphenols are products of the secondary plant metabolism. Polyphenols are of medical interest not only because of their anti-oxidative and anti-inflammatory features [1], but because they are linked to a variety of positive effects including a reduced risk for type 2 diabetes [2], mitigated allergic rhinitis [3], and chronic diseases [4], reduction in plasma cholesterol [5], and an improved formation of nitric oxide [6]. However, a positive correlation between polyphenol content and antioxidant activity has not been found *in vivo* [7,8]. Alleged positive health effects demand for sufficient bioavailability of polyphenols, which depends on different factors, especially the food matrix. Furthermore, polyphenols are mainly present in food as glycosides or polymers, which require hydrolyzation by gut and bacterial enzymes before they can be absorbed [9]. Previous studies on the pharmacokinetics of polyphenols after the consumption of fruit juices, smoothies or puree have shown that up to 20-40% of ingested polyphenols are absorbed in the intestine and thus become bioavailable [10,11]. The percentage of absorption in the colon varies for different groups of polyphenols, with flavan-3-ol derivates (e.g. (epi)-catechin) showing higher rates than quercetin derivates [10]. For dihydrochalcones, several studies have shown that glycosides have to be separated before absorption becomes possible, resulting in low uptake of this polyphenol group [10,12,13].

Apples contain large quantities of polyphenols mainly concentrated in their peel making them promising candidates for food sciences. A number of studies have been conducted to characterize the biochemical composition of apple varieties in order to find varieties with a high content of polyphenolic substances [14-16]. The main polyphenols in apples are flavan-3-ols (Mono-, di-, tri-, and oligomeric), hydroxycinnamic acids, flavonols, dihydrochalcones and anthocyanidins.

Previous studies on polyphenol consumption and metabolism showed large variations between individual test subjects. However, the significance of these findings was limited due to the low sample number of 10 or less subjects. To account for these variations we conducted this current medium scale study. Main objectives were i) to determine time-resolved polyphenol metabolism rates of individual subjects by analyzing both blood and urine samples, and ii) to test, if ingestion of apple juice derived polyphenols influences the antioxidant capacity (AOC) of the blood plasma.

## Materials and methods

### Unfiltered apple juice

The unfiltered apple juice used for this study (*Hasenfit Bio Naturtrüb*) was provided as a single batch by the company Voglsam (Hofkirchen im Traunkreis, Austria). Only apples grown in traditional orchards in the Eferding region of Upper Austria were used for the production of this juice. All apples were grown, harvested and processed under strict organic cultivation rules. The juice was a blend of different apple varieties. In total, four batches of apple juice were tested for their total phenolic content (TPC) and further analyzed by Reversed Phase Chromatography (RPC) for their respective polyphenol composition. The batch containing the highest TPC values was chosen for the study. A total of 80 1 L juice bottles were provided by the company. To verify batch consistency five bottles were chosen at random and their respective polyphenol composition was compared by RPC. Further tests included TPC and sugar content measurements. No significant differences in TPC and composition could be found. Verifying lot consistency ensured that each volunteer ingested identical amounts of polyphenols during the study.

### Study design

The study protocols were approved by the Ethics Commissions of Upper Austria (O2413) and Salzburg (415-EP/73/233-2013), respectively. Study subjects were healthy students of normal weight (i.e. BMI < 25) recruited from the University of Applied Sciences Upper Austria (pool A) and the Paracelsus Medical University Salzburg (pool B). All study participants signed a written informed consent. In total 35 subjects split equally across the two study sites (pool A: 17; pool B: 18) and gender (20 female, 15 male) were approved for the study. Subjects accepted for the study were aged between 19 and 42. During and prior to the study subjects were put on a polyphenol free diet: Volunteers were requested to avoid alcohol and alcoholic beverages, coffee, tea, cocoa, fruits, nuts and vegetables starting 12 hours prior to the study and during the study duration of 10 hours. After an overnight fast, each subject consumed 0.5 L of unfiltered apple juice within 15 minutes. Urine samples were collected from pool A and B subjects prior to and 1, 2, 4, 6, 8 and 10 hours after juice consumption, respectively. In addition, blood samples were taken from pool B subjects prior to and 1, 2, 4 and 6 hours after juice consumption, respectively. Subjects at site B were also asked to keep a dietary record for three days prior to their study participation. Blood samples were processed immediately and extracted plasma was flash-frozen and stored at −20°C until analysis. Urine samples were immediately flash-frozen and stored at −20°C until analysis.

### Sample preparation and extraction

For blood sample collection a standard BD Vacutainer® (Becton Dickinson, Heidelberg, Germany) was used. Plasma was prepared by centrifugation (5,000 g for 5 min) of the blood samples. Urine samples were collected in medical sample collection tubes pre-filled with 1 mL 2 M sodium-phosphate pH 5.8, 0.5 mg ethylene-diaminetetraacetic acid and 20 mg ascorbic acid. Blood and urine samples were extracted using ethyl acetate. 1 mL sample was mixed with 750 μL ethyl acetate and vortexed for 5 minutes. After centrifugation (14,000 g for 5 min) and transfer of the supernatant into a new tube, the remaining solution was extracted for a second time as described. The supernatants were combined and the ethyl acetate was evaporated at 40°C under nitrogen. Pellets were redissolved in 200 μL 20% acetonitrile and analyzed using HPLC-DAD or HPLC-MS. An extraction efficiency of >90% with this method was confirmed by spike recovery tests using commercially available polyphenols (epicatechine, chlorogenic acid, quercetin) at increasing concentrations (0.1 – 100 mg/L).

### Total phenolic content (TPC) measurements

TPC was determined using the Folin-Ciocalteu method [17]. In short, plasma or urine samples were centrifuged at 10,000 rpm for 10 min at room temperature and the supernatant was used for total phenolic quantitation. 1.4 mL deionized water was mixed with 16.7 μL supernatant and 83.3 μL of Folin-Ciocalteu reagent. The mixture stood for 3–6 min at room temperature followed by addition of 167 μL sodium bicarbonate solution (200 g/L). After 70–75 min incubation at room temperature in the dark, absorbance was measured at 750 nm. TPC was expressed as (+)-catechine equivalents in mg/L sample. Each sample was measured in triplicates. For the analysis of the apple juice, the TPC was determined as reported previously with one modification [16]: The samples were centrifuged after carrying out the colorimetric reaction and only the supernatant was measured at 750 nm. This was done to account for the presence of water insoluble oligomeric procyanidins in the unfiltered apple juice.

### Oxygen radical absorbance capacity (ORAC) measurements

Antioxidant capacity of plasma samples was determined using the ORAC assay performed as described previously with slight modifications [18]. Briefly, a mixture of 150 μL fluorescein (10 nM) was used as the target of free radical attack, and 25 μL of 2,2′-azobis(2-amidinopropan)dihydrochlorid (AAPH; 240 mM) was used as a peroxyl radical generator at 37°C combined with 25 μL of each sample or Trolox standard. Trolox standards ranged from 6.25 to 100 μM. 4 mM fluorescein and 1 mM Trolox stock solutions were prepared in 10 mM potassium phosphate buffer pH 7.4 and stored at 2–8°C for up to 3 days. 240 mM 2,2′-azobis(2-amidinopropan)dihydrochlorid stock was prepared using potassium phosphate buffer and used within 10 hours after preparation. Plasma samples were centrifuged (5 min; 10,000 rpm) prior to measurement. Supernatant was further diluted (1:200) with phosphate buffer. Measurements were performed in 96-well plates. In short, 150 μL of fluorescein was pipetted into each well and 25 μL of the standard or diluted sample was added. The plate was incubated at 37°C for 30 min in the dark followed by the addition of 25 μL AAPH solution per well. The decrease in fluorescence of fluorescein was determined by collecting readings at excitation of 485 nm and emission of 520 nm every minute for 90 min on a plate reader (POLARstar omega, BMG LABTECH, Ortenberg, Germany). The ORAC value was calculated using the ORAC plugin of the Omega MARS plate reader software. Each sample was measured in triplicates.

### Trolox equivalent antioxidant capacity (TEAC)

Total antioxidant capacity of blood plasma was measured using the ABTS decolorization assay as previously described [16]. ABTS was dissolved in deionized H_2_O at a concentration of 7 mM and oxidized using 2.45 mM potassium persulfate (final concentration) over night. For analysis, ABTS radical solution was diluted with deionized water to an absorbance of 0.7, measured at 734 nm and equilibrated at 30°C. Plasma samples were diluted 1:10 with deionized water and analyzed by mixing 500 μL of diluted ABTS radical solution with 10 μL of diluted sample. After 5 min incubation at 30°C the absorbance was measured at 734 nm. All samples were analyzed in triplicate. Results were quantified based on a dilution series of Trolox ranging from 6.25 μM to 1 mM. Each sample was measured in triplicates.

### HPLC-DAD and HPLC-MS analysis

HPLC-DAD and HPLC-MS analysis were done as previously described with minor modifications [16]. For reversed phase chromatography (RPC) analysis a Jasco LC-2000 Plus Series system comprising of a quaternary pump with build-in degasser, autosampler, temperature controlled column compartment and diode array detector (DAD) equipped with Chrompass software (all from Jasco Corporation, Tokyo, Japan) was used. Separation was performed on a Hypersil ODS C18 column (250 mm × 4.6 mm inner diameter, 5 μm particle size; Thermo Fisher Scientific, Vienna, Austria). Column temperature was set to 40°C and elution was carried out at 0.8 mL/min. The injection volume for all samples was 20 μL and eluted substances were detected using multiple UV wavelengths from 200 to 350 nm. Catechins, proanthocyanidines and chalcones were detected at 280 nm, hydroxycinnamic acids were detected at 325 nm and flavonols at 360 nm. Using commercial available standards for flavan-3-ols, hydroxycinnamic acids and flavonols individual calibration curves were generated. The limit of detection (LOD) was defined as signal to noise ratio of 2:1 and limit of quantitation (LOQ) as 4:1. For flavan-3-ols LOD of 0.1 mg/L and LOQ of 0.4 mg/L were defined with a linear range of 1–500 mg/L. For hydroxycinnamic acids LOD of 0.05 mg/L and LOQ of 0.2 mg/L were defined with a linear range of 1–1,000 mg/L. For flavonols LOD of 0.1 mg/L and LOQ of 0.3 mg/L were defined with a linear range of 0.1-100 mg/L. Determination for each compound group was semi-quantitative, because individual compounds were quantified based on calibration curves of respective reference substances from each group (epicatechin, 5-coumarylquinic acid, quercetin, 4-hydroxybencoic acid).

For HPLC analysis of polyphenols in the unfiltered apple juice, 1 mL was centrifuged for 10 min at 15,000 rpm followed by 0.1 μm filtration to remove any remaining solids. Verification of individual polyphenols was done by correlating results from mass spectrometry analysis with the UV spectra and retention times of reference substances. Quantitation of identified polyphenols was done using UV absorption by reference substances of known concentrations prepared in deionized water. For HPLC analysis of plasma and urine samples were extracted as described before. In total 18 flavan-3-ols, 8 hydroxycinnamic acids, 12 flavonols and 8 benzoic acids were identified by LC-MS and quantified using UV absorption of reference substances for each group.

The following conditions were used for HPLC-DAD analysis: Mobile phase A contained 0.1% trifluoroacetic acid in water. Mobile phase B contained 80% acetonitrile and 0.1% trifluoroacetic acid. The starting conditions were 97.5% A and 2.5% B. Elution was performed with a linear gradient: The proportion of B was increased to 10% at 20 min, 20% at 32 min and 50% at 45 min. Sanitation was done using 100% B for 5 min followed by re-equilibration with starting conditions for 5 min.

HPLC-MS analyses were performed on an Agilent 1100 HPLC system equipped with a vacuum degasser, a quaternary pump, an autosampler and an UV–vis diode array detector (all from Agilent Technologies, Santa Clara, USA). Separations were carried out using an ODS Hypersil column (250 mm × 4.6 mm inner diameter; 1.8 μm particle size; Thermo Fisher Scientific, Austria). Analytes were separated by gradient elution with 0.1% (v/v) formic acid (A) and acetonitrile containing 0.1% (v/v) formic acid (B) at a flow-rate of 1 mL/min. The starting conditions were 97.5% A and 2.5% B. The proportion of B was increased to 10% at 20 min, 20% at 32 min, 50% at 45 min and 80% at 50 min. Sanitation was done using 20% A, 80% C for 10 min followed by re-equilibration with starting conditions for 10 min. The column was thermostated at 40°C and the injection volume was 20 μL.

For sugar analysis of the apple juice a Jasco RI-2031 Plus detector (Jasco Corporation, Tokyo, Japan) was used. Separation was performed on an Aminex HPX-87 H300 carbohydrate column (300 mm × 7.8 mm inner diameter, 9 μm particle size, BIO-RAD, Hercules, USA). Column temperature was set to 80°C and isocratic elution was carried out at 0.8 mL/min. As mobile phase 5 mM sulfuric acid in ddH_2_O was used. The injection volume for all samples was 20 μL and eluted sugars were detected by refractive index. Limit of detection (LOD) was defined as signal-to-noise ratio of 2:1 and limit of quantitation (LOQ) as 4:1. LOD was 10 mg/L and 20 mg/L for glucose; and 1 mg/L and 5 mg/L for fructose, respectively.

MS measurements were done on a 6520 quadrupole/time-of-flight instrument equipped with an electrospray ionization source (Agilent Technologies, Santa Clara, USA). Results were obtained using the following settings: MS capillary voltage 3750 V, fragmentor voltage 180 V, drying-gas (nitrogen) flow rate 12 L/min, drying-gas temperature 350°C, and nebulizer pressure 60 psi. Scanning mass range was from *m/z* 70–3200 with an acquisition rate of 1.0 spectra/s in the negative MS mode.

### Statistics

Results were obtained from three independent analyses (mean ± SD). MS Office Professional Plus 2010 (v 14.0.7128.5000, Microsoft Corporation) was used for data compilation and statistical evaluation (Excel data analysis add-in, Microsoft Corporation). Significance testing was done using GraphPad Prism 6 for Windows software package (GraphPad Software Inc.). Differences were considered significant with p ≤ 0.05 or p ≤ 0.01 using *t*-test and two-way ANOVA grouped analysis tests.

## Results

### Biochemical characterization of unfiltered apple juice

The apple juice “Hasenfit Bio Naturtrüb” used for this study was supplied by a local producer and was prepared from organically grown apples only. The juice selected for the study had a total phenolic content (TPC) of more than 2,160 mg/L. By drinking 500 mL apple juice each subject ingested about 1,080 mg of polyphenols. The water soluble phenolic composition of the used apple juice analyzed by reversed phase chromatography (RPC) is given in Additional file 1: Table S1. The respective RPC chromatogram is shown in Additional file 1: Figure S1. Unlabeled peaks were not identified during the HPLC-MS analysis. The apple juice contained large quantities of flavan-3-ols and hydroxycinnamic acids. Flavonols were found only in small amounts. However, as RPC analysis requires the removal of solid particles, all water-insoluble substances including polyphenols of higher molecular weight, e.g. oligomeric procyanidins (OPCs) cannot be detected by this method [19]. Nevertheless, these substances were present in the juice as the TPC of the centrifuged sample was significantly lower than the one measured with the unfiltered apple juice prior to centrifugation (data not shown). The selected apple juice contained large amounts of sugar, with about 26.4 g/L glucose and 79.0 g/L fructose (Additional file 1: Table S1). Consequently, by drinking the 500 mL juice each study subject consumed about 13 g of glucose and 40 g of fructose.

### Changes in total phenolic content of plasma after apple juice consumption

Polyphenols in blood plasma samples were determined by the Folin-Ciocalteu (FC) method. The averaged TPC value in the overnight fast samples was found to be 1,560 mg/L (Table 1) and showed a steady increase after juice consumption. After six hours the average TPC level was found to be 1,700 mg/L, corresponding to a significant total increase of about 10% (1-way ANOVA, p < 0.003). Polyphenols are absorbed by the gut as aglycones and subsequently released into the blood stream only after chemical modification (e.g. methylation, sulfation and glucuronidation) changing their chemical properties [20]. Thus, calculation of absorption rates is not possible, but the observed 10% increase in TPC level indicates that significant amounts of the polyphenols ingested with the apple juice were taken up by the individual subjects within the monitored time frame.

**Table 1:** **Overview of average (± SD), minimal and maximal values for total phenolic content and RPC analysis in plasma samples**

	Total phenolic content [mg/L]				
	o/n	1 h	2 h	4 h	6 h
Average	1,559 ± 167	1,575 ± 165	1,621 ± 139	1,676 ± 168	1,706 ± 167
Min	1.025	1.121	979	1.188	1.145
Max	1.749	1.66	1.698	1.853	1.779
	**Reversed phase chromatography [mg/L]**				
	**o/n**	**1 h**	**2 h**	**4 h**	**6 h**
Average	6 ± 5	4 ± 3	4 ± 3	4 ± 4	7 ± 5
Min	0	0	0	0	0
Max	21	12	12	14	15

**Figure 1:**
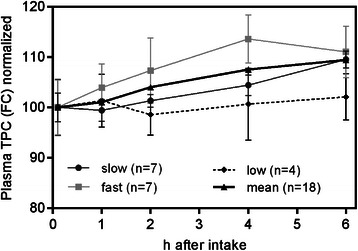
**Phenolic content in plasma samples determined by Folin Ciocalteu (FC) normalized to the overnight fast sample and metabolism rates.** Significant differences in metabolism rates between subjects grouped according to their respective maxima in TPC could be observed. Values are means ± SD (n = 18).

Analysis of the individual subjects led to the identification of large variations in the TPC levels. Concentrations in the overnight fast samples ranged from 1,025 to 1,749 mg/L, a variation of about 64% (Table 1). These differences remained constant during the course of the study. When analyzing the plasma TPC levels of individual subjects at single time points, it became apparent that the observed changes in total phenolics were highly variable between individuals: We categorized the subjects into three groups, according to the time of highest polyphenol content during the study. The majority of the subjects showed maximum polyphenol concentrations between four to six hours after juice consumption (Figure 1, “slow” and “fast” response). Four subjects (“low”) showed hardly any detectable change within the study period. Observed variations in polyphenol content between the three assigned groups were highly significant (2-way ANOVA; p < 0.01 at four hours).

### Pharmacokinetic profiles of apple juice polyphenols in plasma samples

After ingestion, different polyphenols were detected and quantified using HPLC-DAD. For this, a total of 46 peaks were assigned by HPLC-MS to four polyphenol groups: flavan-3-ols, flavonols, hydroxycinnamic acids and benzoic acids (Additional file 1: Table S1 and Figure S2). Precise structural-chemical analysis was not performed due to lack of reference material of modified polyphenols and quantification was therefore only semi-quantitative. Peaks that could not be assigned to those groups were excluded from the analysis (about 30% of total area). It is important to point out that the extraction procedure results in a loss of all protein-bound molecules (for example serum albumin is known for unspecific binding of phenolic compounds) [21,22]. Consequently, only free molecules were analyzed, resulting in apparent low levels of polyphenols detected in the plasma samples by HPLC analysis. The vast majority of detected substances belonged to the flavan-3-ol group, which was present in a concentration range of 1–20 μmol/L. Free flavonols were found only in a minority of subjects at concentrations around 1 μmol/L.

Similar to the results from FC measurements, RPC analysis showed large variations of the polyphenol levels between individual subjects in overnight fast samples from below the detection limit to more than 20 μg/mL (Table 1). When comparing the average concentration of free polyphenol compounds after six hours to the overnight fast samples, a 19% increase in the total content could be observed.

### Urinary excretion of polyphenols

Urine samples were collected directly before and at different times within ten hours post apple juice consumption and analyzed for their polyphenolic content. The average TPC level for the overnight-fast samples was about 770 mg/L (Table 2). Similar to the plasma samples, individual subjects showed large variations, with total phenolic concentration varying between 130 and 2,300 mg/L (about 19-fold). Within the first two hours, the average TPC decreased to 360 mg/L, and increased significantly reaching a maximum of 530 mg/L after six hours (about 47% increase compared to the lowest content two hours after juice consumption; p ≤ 0.02), before decreasing again to 470 mg/L after ten hours. Comparable to the overnight fast samples, after six hours the phenolic contents varied remarkably between 120 and 2,160 mg/L for the subjects with the lowest and highest concentration, respectively. The subjects with the highest and lowest overnight fast TPC showed also the highest and lowest contents after six hours. Subjects defined as a “low response population” were characterized by small variations and no detectable increase in their polyphenol excretion within the time frame of the study (Table 2). On the other hand, the five subjects with the highest TPC values in the overnight fast samples showed a large decrease within two hours from 1,050 to 360 mg/L, followed by a massive increase in phenolic excretion six hours after juice consumption, with a mean phenolic excretion of about 1,400 mg/L (4-fold increase).

**Table 2:** **A. Overview of average (± SD), minimal and maximal values for total phenolic content and RPC analysis in urine samples; B. Averaged TPC values in mg/L ± SD of the five subjects with lowest and highest overnight content and their respective time responses**

A	Total phenolic content [mg/L]						
	o/n	1 h	2 h	4 h	6 h	8 h	10 h
Average	772 ± 442	493 ± 324	362 ± 216	455 ± 254	534 ± 403	487 ± 251	467 ± 193
Min	127	323	199	194	126	174	179
Max	2.277	984	418	709	2.163	1.217	672
	**Reversed phase chromatography [mg/L]**						
	**o/n**	**1 h**	**2 h**	**4 h**	**6 h**	**8 h**	**10 h**
Average	94 ± 85	48 ± 63	28 ± 41	35 ± 53	34 ± 28	37 ± 40	42 ± 34
Min	2	1	0	1	2	1	6
Max	434	365	226	294	107	163	120
**B**	**Total phenolic content [mg/L]**						
	**o/n**	**1 h**	**2 h**	**4 h**	**6 h**	**8 h**	**10 h**
No response group	220 ± 33	204 ± 44	206 ± 39	226 ± 30	223 ± 34	247 ± 31	219 ± 15
Max response group	1,052 ± 260	805 ± 104	366 ± 42	826 ± 84	1,375 ± 200	885 ± 121	672 ± 59

**Figure 2:**
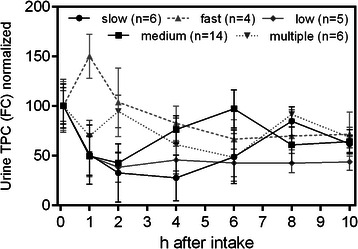
**Phenolic content determination in urine samples determined by Folin Ciocalteu (FC) normalized to the overnight fast sample.** Marked differences in metabolism rates between individual subjects could be observed. Using their respective maxima in TPC values study subjects could be grouped into 5 distinct groups. Values are means ± SD (n = 35).

Similar to the classification of the subjects based on their plasma phenolic compound concentrations, urine analysis allowed for a categorization of subjects into different groups according to the time-point of maximal polyphenol excretion. The normalized TPC profiles for each group are shown in Figure 2. Six subjects showed maximum excretion eight hours after ingestion (“slow”), 14 after six hours (“average”), and four after one hour (“fast”). Six subjects showed two maxima, one hour and 6–8 hours after apple juice consumption (“multiple”). Finally, five subjects (“low”) displayed no significant increase in polyphenol excretion at all. 2-way ANOVA analysis of the averaged contents of each group for each time point showed these differences being significant with p < 0.001 at their respective maxima.

Polyphenols present in urine were analyzed by RPC as well. The majority of the detected polyphenols belonged to the flavan-3-ol and benzoic acid classes. Only small amounts of hydroxycinnamic acids could be detected in the urine samples, and flavonols could only be found in the urine of two subjects. When focusing on the time resolved excretion of the averaged study contingent, the concentration of phenolic degradation products detected by RPC decreased to a minimum two hours after juice consumption, and increased constantly from then on till the end of the study period (Table 2). The RPC results are in good agreement with those from the FC measurements and the same classification in the different categories was possible (data not shown).

When comparing data derived from FC and RPC analysis, identical curve progressions for both methods could be identified. Thus, results obtained from FC measurements could be verified by the RPC quantification of individual phenolic compounds. The overall excretion of phenolic compounds in the urine determined by RPC ranged between 0.6 and 93.4 mg, with an average of 14.8 mg. Taken together, our results indicate great variations between individual subjects with respect to pharmacokinetics and metabolism rates of phenolic substances.

### Comparison of normalized phenolic contents in plasma and urine samples

When we compared plasma and urine samples, changes in phenolic contents were found to be temporally delayed in the urine samples. After juice consumption the mean TPC of all plasma samples showed an immediate increase (within one hour). For urine samples, the observed increase in TPC was delayed for 2–3 hours starting around 3–4 hours after juice consumption. Similar to the results obtained from TPC analysis, RPC measurements showed an offset in phenolic contents increase between plasma and urine samples by 2–3 hours.

**Figure 3:**
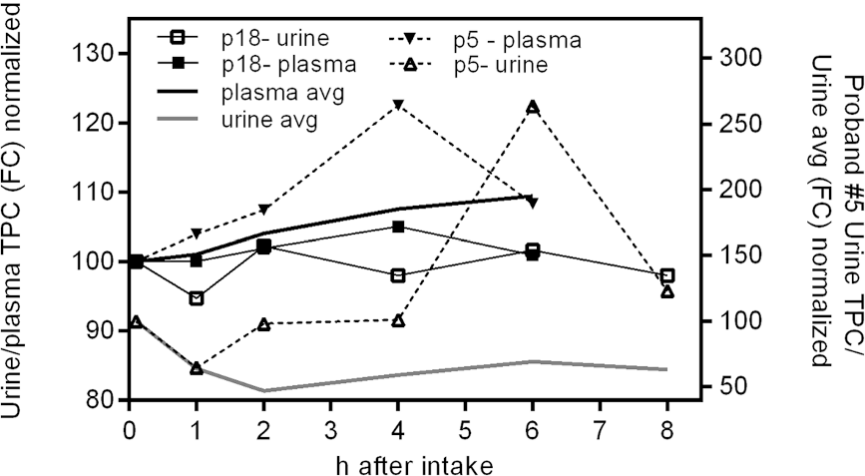
**Comparison of time dependent polyphenol content in urine and plasma samples of individual subjects determined by the Folin Ciocalteu (FC) method and normalized to the overnight fast sample.** Subject #05 showed strong maxima after 4 hours in plasma and 6 hours in urine samples whereas subject #06 showed no observable maxima in both plasma and urine samples over the whole study period. The overall averaged curves for the study cohort are included for comparison reasons.

In Figure 3 the time course of plasma and urine TPC samples for subjects #5 and #18 are illustrated to show the two extremes observed within our study subjects in comparison to the averaged results. In subject #5, the TPC level in plasma was found to increase one to two hours after apple juice consumption and continued to slightly rise until it peaked after four hours. For this subject, the urine samples showed no change within the first two hours; an increase could be observed starting after three to four hours followed by a maximum concentration six hours after juice consumption. The same pattern was observed in the RPC analysis. In contrast, subject #18 showed no observable increase in polyphenol levels within the studied time frame at all. The TPC levels in both urine and plasma samples remained constant during the whole study time and showed only about 5% variation.

### Changes in antioxidant capacity of plasma samples as a result of juice consumption

**Figure 4:**
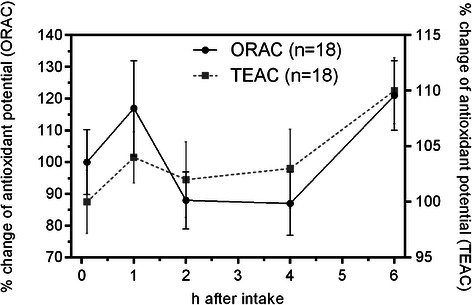
**Antioxidant capacity (AOC) of plasma samples in the course of the study determined by the Oxygen Radical Absorbance Capacity (ORAC) and Trolox Equivalent Antioxidant Capacity (TEAC) assays.** Relative change of antioxidant capacity was calculated for the averaged values of each time point against the overnight fast sample. Values are means ± SD (n = 18).

It is still not completely clear whether polyphenolic substances present in the diet can positively influence the AOC *in-vivo* [9]. To address this question we determined the AOC of plasma samples obtained during this study by the Oxygen Radical Absorbance Capacity (ORAC) and Trolox Equivalent Antioxidant Capacity (TEAC) assays. As shown in Figure 4 the mean AOC determined by the ORAC method was found to increase by about 17% after one hour. However, it dropped significantly (about 13% compared to time zero) within two hours. Interestingly, it increased again six hours after the start of the study. These results were confirmed by the TEAC assay. A similar trend was not detected in either TPC or RPC analysis. Thus, a correlation of polyphenolic substances present in the apple juice and an increase in AOC of blood plasma was not established.

### Comparison of renal excretion and plasma concentrations in female and male subjects

**Figure 5:**
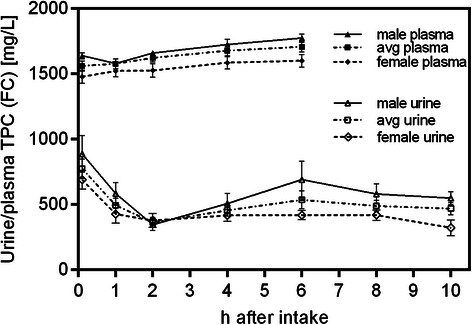
**Comparison of total averaged results to the individual female and male subject groups.** Plasma and urine samples were analyzed by Folin Ciocalteu (FC) measurements. Values are means ± SD (n = 4 for male, n = 14 for female subjects in plasma; n = 15 for male, n = 20 for female in urine).

In total 20 female and 15 male subjects participated in this study. The mean TPC of the urine samples from female subjects at time zero was found to be significantly lower compared to the averaged male subjects (700 mg/L versus 900 mg/L, p < 0.001). This also holds true for plasma TPC levels, although with less significance (p < 0.01) due to the lower number (n = 4) of plasma samples from male volunteers. The differences in TPC values remained constant over the study duration (p < 0.01 to p < 0.001) with the exception of the 1 hour time point for plasma and 2 hour time point for urine, respectively. The changes in TPC levels during the course of the study showed similar characteristic in both genders with nearly identical curve progressions (Figure 5).

## Discussion

Apples are an integral part of daily fruit intake in the Western world, and provide a major source of polyphenolic substances in daily life. Flavonoids and hydroxycinnamic acids present in large quantities in apple peel and flesh receive particular interest for various health-benefiting effects on the human body [23]. However, as they cannot be synthesized by the human body their half-life is low. In the work presented here, a medium-sized group of young healthy middle European adults consumed unprocessed apple juice. Blood and urine samples were collected before and at different time points up to 10 hours post apple juice consumption and analyzed for polyphenol content, to evaluate the individual polyphenol metabolism for each subject.

To our knowledge, this study for the first time describes diverse polyphenol metabolism patterns in humans with respect to dietary polyphenol uptake. Differences in the metabolization speed could be seen in both blood plasma and urine samples: Some subjects were found to excrete the ingested phenolic compounds in a shorter period of time than others required for uptake in the gut. Similar findings have been observed before. For example, in a previous study on apple juice polyphenol metabolism, single subjects were identified that showed more than 2-fold slower transportation of polyphenols through the colon [10], consistent with a substantially delayed detection of polyphenols in the respective plasma and urine samples. In our study we found that 14% of the subjects showed no apparent metabolism within the course of the study. It can be assumed that for a significant part of the general population the polyphenol turnover is slower than 8–10 hours. This has been reported (at least partially) in a previous study, where one out of 5 analyzed subjects showed such delayed polyphenol metabolism kinetics [11]. However, due to the low abundance of these individuals they were dropped out as single outsiders [10,11]. The uptake of polyphenols does not only depend on the individual gut micro-flora, but on genetic traits as well. For example, a recent study on the effects of green coffee beans on *Nrf2* signaling provided data on possible genotypic differences influencing the efficacy of food constituents including polyphenols in humans [24]. It is feasible that such differences in the metabolization of polyphenols have an impact on overall beneficial effects of a polyphenol rich diet in these persons. Consequently, it would be of great interest to focus on such subjects for additional analysis, e.g. higher incidents of cancer types that are thought to be affected by polyphenols or higher prevalence for cardiovascular diseases. Another factor that has to be taken into consideration is the observation that the absorption of polyphenols is influenced by individual differences including the length of the small intestine, the microbiome or genetic factors [25]. In the present study the majority (40%) of all subjects was characterized by a mean excretion maximum about six hours after apple juice consumption, being in good agreement with previous studies on unfiltered apple juice [11], green tea [26], or whole apple puree [6]. The remaining subjects distributed equally into groups with fast or slow metabolism kinetics and a group characterized by two polyphenol content maxima.

Absorption of different polyphenol groups in the intestine and consequently their bioavailability has been reported to differ significantly [10,11]. For example, anthocyanins are rarely detected in blood or urine samples, whereas for flavonols and flavonols recoveries of 1-30% were reported. These depended on dosage, the sample matrix and experimental methodologies, such as analysis of parent compounds or metabolites. We found an average urinary excretion of 2.5% of the ingested polyphenols, which is in agreement with those recovery values. However, as some groups of polyphenols are known to be excreted in bile this value is probably a significant underestimation of the actual absorption [27].

When comparing female and male subjects we found lower polyphenol concentrations in the samples from female subjects. Also, the maximum polyphenol peak concentration in urine samples was reduced in the female group. However, the observed relative increases in blood plasma and urine were similar for both genders. In order to study such correlations in detail and describe general gender trends, a much larger study contingent will be required.

A key biological effect of polyphenols is their antioxidant activity. Although *in-vitro* experiments clearly approved the positive influence of polyphenols on oxidative stress, the effects of these substances in living systems are inconclusive or could not confirm *in-vitro* data at all [8,28]. We found a significant increase of the plasma AOC within one hour after apple juice consumption. However, this increase could not be explained by apple juice derived polyphenols: While the mean plasma AOC was found to increase by about 17% within one hour post apple juice consumption, the plasma TPC levels were only elevated by 1% within the same time period. This finding is in agreement with a study presented by Lotitio *et al*. [29]. In this work the authors were able to corroborate the increase in plasma AOC to high urate concentrations being a by-product of the metabolism of fructose. The observed spike in AOC in our study can similarly be explained by the 40 g fructose and 13 g glucose ingested by the subjects. The subsequent increase after six hours can be explained likewise, as subjects consumed a meal after four hours that was free of polyphenols, but included carbohydrates. Taken together our data suggest that the observed increase in AOC is not the result of apple polyphenol absorption.

## Conclusions

While analyzing the uptake and excretion of apple juice derived polyphenols in a medium-sized population, the existence of discrete sub-populations within the study subjects was observed. Although the majority of subjects displayed expected metabolism time responses, a small but significant number of subjects showed either very fast or very slow pharmacokinetics. Further studies are needed to determine whether the polyphenol concentration in subjects with very delayed absorption is sufficient to positively influence cellular functions, e.g. the inhibition of growth factor receptors [30] or the reduction of the blood glucose and cholesterol levels [31]. As apple polyphenols did not influence the AOC, future studies should focus more on other possibilities how polyphenols accomplish the health benefits attributed to them.

## Additional file

Additional file 1: Table S1. Biochemical composition of Hasenfit Bio Naturtrüb apple juice used for the study. Individual polyphenols were identified by HPLC-MS. Concentration of glucose and fructose was quantified by HPLC. Total phenolic content was determined by Folin Ciocalteu (FC) measurements. **Table S2.** Overview of 47 polyphenols selected for characterization of extracted samples in plasma and urine samples. Substances were identified by HPLC-MS. **Figure S1.** HPLC elution profile of apple juice. HPLC elution profile of Hasenfit Bio Naturtrüb apple juice used for the study detected at 280 nm, 320 nm and 360 nm, respectively. 1, Procyanidin B1; 2, Epigallocatechin; 3, Chlorogenic acid; 4, Procyanidin B2; 5, Caffeic acid; 6, Epicatechin; 7, 4-p-Coumarylquinic acid; 8, Epicatechingallate; 9, Phloretin 2′-O-xylosyl-glucoside; 10, Phloridzin; 11, Quercetin 3-O-rutinoside; 12, Quercetin 3-O-galactoside; 13, Quercetin; 14, Quercetin 3-O-xyloside; 15, Quercetin 3-O-rhamnoside. **Figure S2.** HPLC elution profile of urine sample. HPLC elution profile of a representative extracted urine sample detected at 280 nm, 320 nm and 360 nm, respectively. Peaks used for analysis are labeled with 1–47. In total 18 flavan-3-ols, 8 hydroxycinnamic acids, 12 flavonols and 8 benzoic acids were used for analysis.

## Abbreviations

AOC: Antioxidant capacity
DAD: Diode array detector
FC: Folin-Ciocalteu
HPLC: High performance liquid chromatography
LOD: Level of detection
LOQ: Level of quantification
MS: Mass spectrometry
OPC: Oligomeric procyanidins
ORAC: Oxygen radical absorbance capacity
RPC: Reversed phase chromatography
TEAC: Trolox equivalent antioxidant capacity
TPC: Total phenolic content
