# Supplementary material for: Differences in pharmacokinetics of apple polyphenols after standardized oral consumption of unprocessed apple juice
Source: Nutr J. 2015 Apr 1;14:32. doi: 10.1186/s12937-015-0018-z (PMC4396834; doi:10.1186/s12937-015-0018-z)
Supplement: Additional file 1: Table S1. — Biochemical composition of Hasenfit Bio Naturtrüb apple juice used for the study. Individual polyphenols were identified by HPLC-MS. Concentration of glucose and fructose was quantified by HPLC. Total phenolic content was determined by Folin Ciocalteu (FC) measurements. Table S2. Overview of 47 polyphenols selected for characterization of extracted samples in plasma and urine samples. Substances were identified by HPLC-MS. Figure S1. HPLC elution profile of apple juice. HPLC elution profile of Hasenfit Bio Naturtrüb apple juice used for the study detected at 280 nm, 320 nm and 360 nm, respectively. 1, Procyanidin B1; 2, Epigallocatechin; 3, Chlorogenic acid; 4, Procyanidin B2; 5, Caffeic acid; 6, Epicatechin; 7, 4-p-Coumarylquinic acid; 8, Epicatechingallate; 9, Phloretin 2′-O-xylosyl-glucoside; 10, Phloridzin; 11, Quercetin 3-O-rutinoside; 12, Quercetin 3-O-galactoside; 13, Quercetin; 14, Quercetin 3-O-xyloside; 15, Quercetin 3-O-rhamnoside. Figure S2. HPLC elution profile of urine sample. HPLC elution profile of a representative extracted urine sample detected at 280 nm, 320 nm and 360 nm, respectively. Peaks used for analysis are labeled with 1–47. In total 18 flavan-3-ols, 8 hydroxycinnamic acids, 12 flavonols and 8 benzoic acids were used for analysis. [file 12937_2015_18_MOESM1_ESM.docx]

**Additional file 1**

**Table S1**. Biochemical composition of Hasenfit Bio Naturtrüb apple juice used for the study. Individual polyphenols were identified by HPLC-MS. Concentration of glucose and fructose was quantified by HPLC. Total phenolic content was determined by Folin Ciocalteu (FC) measurements.

| **Composition of apple juice** | **mg/L** |  | **Sugar content:** | |
| --- | --- | --- | --- | --- |
|  |  |  |  | |
| **Flavan-3-ols** | |  | 26.4 g/L Glucose | |
|  |  |  | 79.0 g/L Fructose | |
| Procyanidin B1 | 22.3 |  | **Total phenolic content:** | |
| Procyanidin B2 | 106.5 |  |  | |
| Epicatechine | 72.7 |  | 2,150 mg/L | |
| Epicatechingallate | 12.6 |  |  |  |
| Phloretin 2'-O-xylosyl-glucoside | 28.9 |  |  |  |
| Phloridzin | 9 |  |  |  |
|  |  |  |  |  |
| **Flavonols** | |  |  |  |
|  |  |  |  |  |
| Quercetin 3-O-rutinoside | 0.2 |  |  |  |
| Quercetin 3-O-galactoside | 0 |  |  |  |
| Quercetin | 0.7 |  |  |  |
| Quercetin 3-O-xyloside | 0.9 |  |  |  |
| Quercetin 3-O-rhamnoside | 1.9 |  |  |  |
|  |  |  |  |  |
| **Hydroxycinnamic Acids** | |  |  |  |
|  |  |  |  |  |
| Chlorogenic acid | 337.2 |  |  |  |
| 4-p-Coumaroylquinic acid | 32.5 |  |  |  |
| 3,4-Dihydroxyhydrocinnamicacid | 2.2 |  |  |  |

**Table S2**. Overview of 47 polyphenols selected for characterization of extracted samples in plasma and urine samples. Substances were identified by HPLC-MS.

| **Peak #** | **Substance class** |  | **Peak #** | **Substance class** |
| --- | --- | --- | --- | --- |
| 1 | Benzoic Acid |  | 24 | Flavan-3-ol |
| 2 | Benzoic Acid |  | 25 | Hydroxycinnamic Acids |
| 3 | Flavan-3-ol |  | 26 | Hydroxycinnamic Acids |
| 4 | Flavan-3-ol |  | 27 | Benzoic Acid |
| 5 | Flavan-3-ol |  | 28 | Flavan-3-ol |
| 6 | Benzoic Acid |  | 29 | Flavonol |
| 7 | Flavan-3-ol |  | 30 | Flavan-3-ol |
| 8 | Flavan-3-ol |  | 31 | Hydroxycinnamic Acids |
| 9 | Flavan-3-ol |  | 32 | Flavan-3-ol |
| 10 | Flavan-3-ol |  | 33 | Flavan-3-ol |
| 11 | Flavonol |  | 34 | Flavonol |
| 12 | Hydroxycinnamic Acids |  | 35 | Benzoic Acid |
| 13 | Flavan-3-ol |  | 36 | Flavan-3-ol |
| 14 | Benzoic Acid |  | 37 | Flavonol |
| 15 | Flavan-3-ol |  | 38 | Benzoic Acid |
| 16 | Hydroxycinnamic Acids |  | 39 | Flavonol |
| 17 | Hydroxycinnamic Acids |  | 40 | Flavonol |
| 18 | Flavan-3-ol |  | 41 | Flavonol |
| 19 | Flavan-3-ol |  | 42 | Flavonol |
| 20 | Flavan-3-ol |  | 43 | Flavonol |
| 21 | Hydroxycinnamic Acids |  | 44 | Flavonol |
| 22 | Benzoic Acid |  | 45 | Flavonol |
| 23 | Hydroxycinnamic Acids |  | 46 | Benzoic Acid |
|  |  |  | 47 | Flavonol |

**Figure S1**: HPLC elution profile of apple juice.


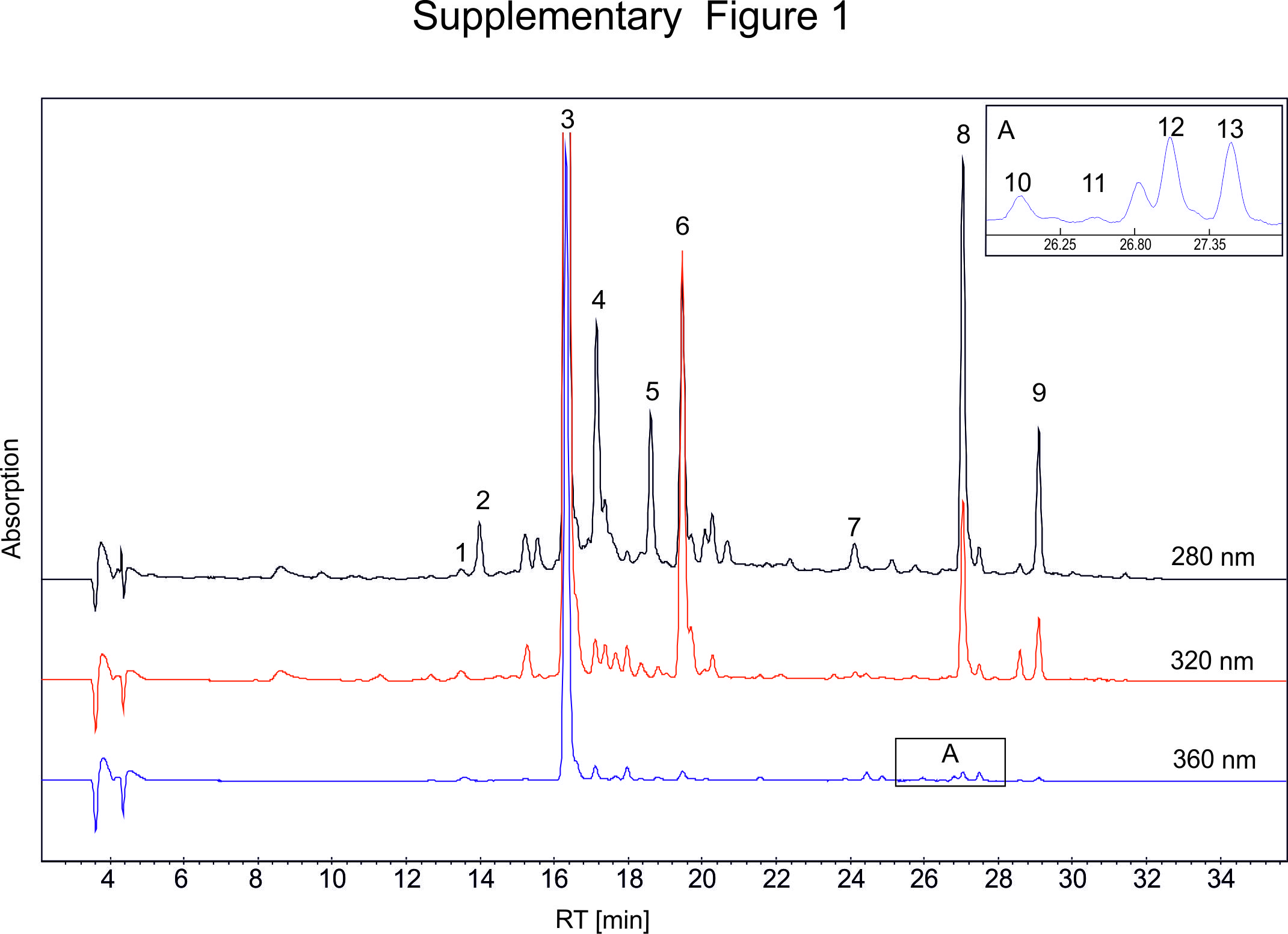


HPLC elution profile of Hasenfit Bio Naturtrüb apple juice used for the study detected at 280 nm, 320 nm and 360 nm, respectively. 1, Procyanidin B1; 2, Epigallocatechin; 3, Chlorogenic acid; 4, Procyanidin B2; 5, Caffeic acid; 6, Epicatechin; 7, 4-p-Coumarylquinic acid; 8, Epicatechingallate; 9, Phloretin 2'-O-xylosyl-glucoside; 10, Phloridzin; 11, Quercetin 3-O-rutinoside; 12, Quercetin 3-O-galactoside; 13, Quercetin; 14, Quercetin 3-O-xyloside; 15, Quercetin 3-O-rhamnoside.

**Figure S2**: HPLC elution profile of urine sample.


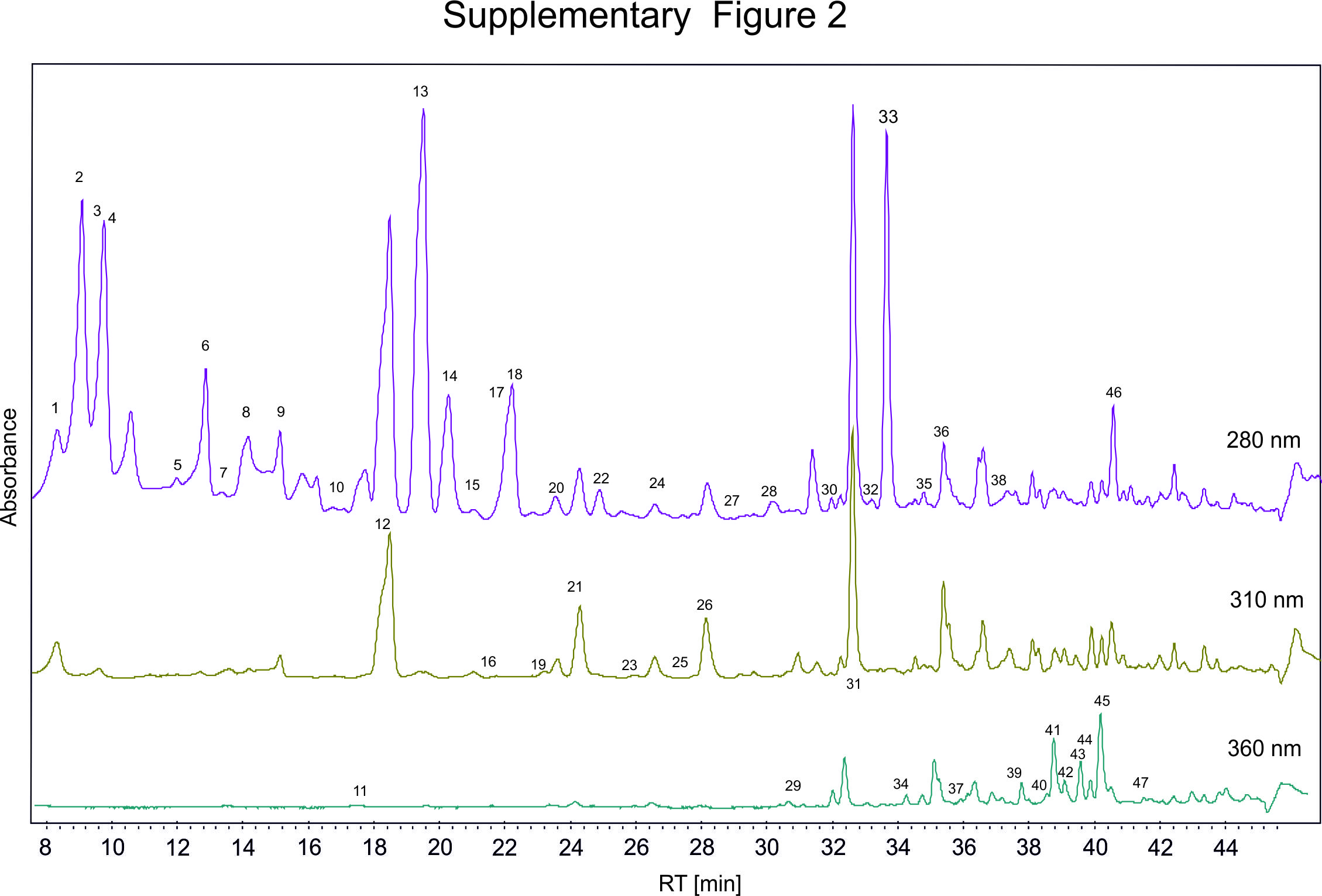


HPLC elution profile of a representative extracted urine sample detected at 280 nm, 320 nm and 360 nm, respectively. Peaks used for analysis are labeled with 1-47. In total 18 flavan-3-ols, 8 hydroxycinnamic acids, 12 flavonols and 8 benzoic acids were used for analysis.
